# Protein-enriched meal replacements do not adversely affect liver, kidney or bone density: an outpatient randomized controlled trial

**DOI:** 10.1186/1475-2891-9-72

**Published:** 2010-12-31

**Authors:** Zhaoping Li, Leo Treyzon, Steve Chen, Eric Yan, Gail Thames, Catherine L Carpenter

**Affiliations:** 1 Center for Human Nutrition, David Geffen School of Medicine at UCLA, Los Angeles, CA 90095, USA; 2 Department of Medicine, Greater Los Angeles VA Health Care System, Los Angeles, CA 90073, USA

## Abstract

**Background:**

There is concern that recommending protein-enriched meal replacements as part of a weight management program could lead to changes in biomarkers of liver or renal function and reductions in bone density. This study was designed as a placebo-controlled clinical trial utilizing two isocaloric meal plans utilizing either a high protein-enriched (HP) or a standard protein (SP) meal replacement in an outpatient weight loss program.

**Subjects/methods:**

100 obese men and women over 30 years of age with a body mass index (BMI) between 27 to 40 kg/m^2 ^were randomized to one of two isocaloric weight loss meal plans 1). HP group: providing 2.2 g protein/kg of lean body mass (LBM)/day or 2). SP group: providing 1.1 g protein/kg LBM/day. Meal replacement (MR) was used twice daily (one meal, one snack) for 3 months and then once a day for 9 months. Body weight, lipid profiles, liver function, renal function and bone density were measured at baseline and 12 months.

**Results:**

Seventy subjects completed the study. Both groups lost weight (HP -4.29 ± 5.90 kg vs. SP -4.66 ± 6.91 kg, p < 0.01) and there was no difference in weight loss observed between the groups at one year. There was no significant change noted in liver function [AST (HP -2.07 ± 10.32 U/L, p = 0.28; SP 0.27 ± 6.67 U/L, p = 0.820), ALT (HP -1.03 ± 10.08 U/L, p = 0.34; SP -2.6 ± 12.51 U/L, p = 0.24), bilirubin (HP 0.007 ± 0.33, U/L, p = 0.91; SP 0.07 ± 0.24 U/L, p = 0.120), alkaline phosphatase (HP 2.00 ± 9.07 U/L, p = 0.240; SP -2.12 ± 11.01 U/L, p = 0.280)], renal function [serum creatinine (HP 0.31 ± 1.89 mg/dL, p = 0.380; SP -0.05 ± 0.15 mg/dL, p = 0.060), urea nitrogen (HP 1.33 ± 4.68 mg/dL, p = 0.130; SP -0.24 ± 3.03 mg/dL, p = 0.650), 24 hour urine creatinine clearance (HP -0.02 ± 0.16 mL/min, p = 0.480; SP 1.18 ± 7.53 mL/min, p = 0.400), and calcium excretion (HP -0.41 ± 9.48 mg/24 hours, p = 0.830; SP -0.007 ± 6.76 mg/24 hours, p = 0.990)] or in bone mineral density by DEXA (HP 0.04 ± 0.19 g/cm^2^, p = 0.210; SP -0.03 ± 0.17 g/cm^2^, p = 0.320) in either group over one year.

**Conclusions:**

These studies demonstrate that protein-enriched meals replacements as compared to standard meal replacements recommended for weight management do not have adverse effects on routine measures of liver function, renal function or bone density at one year. Clinicaltrial.gov: NCT01030354.

## Background

Obesity and overweight have reached epidemic proportions in the U.S. and increasingly around the world [1,2]. A number of studies have suggested that protein is the most satiating macronutrient and promotes the retention of lean body mass. Meals with increased protein to carbohydrate ratios have been demonstrated to increase satiety and decrease food intake [3,4] by comparison to standard protein intake. Increased protein intake results in both improved weight loss and improved maintenance of weight loss [5,6]. Therefore, protein-enriched or supplemented meal replacements have found their way into weight management practice.

There has been some concern that the long-term use of high protein diets may damage liver function, renal function, or reduce bone density [7,8]. While there are studies of the effects of increased intake of animal protein in the diet, protein-enriched meal replacements have not been evaluated in comparison to standard meal replacements in terms of effects on liver function, renal function, and bone mineral density in free-living populations.

Meal replacement (MR) is an important strategy in designing structured diets for weight management due to their simplicity, low cost, and convenience of protein-enriched meal replacement shakes by comparison to fast food meals [5,9,10]. Noakes et al [11] have shown that meal replacements are as effective as structured weight-loss diets. MR simplifies the weight loss plan by replacing one or two meals a day with a product of defined nutrient and calorie content. MR leads to increased weight losses over twelve weeks compared to simply restricting the intake of favorite food, and weight losses have been shown to be maintained for up to 4 years with the inclusion of one MR per day [12]. The present study was designed to recommend isocaloric weight management programs through the inclusion of either a protein or a carbohydrate supplement to a standard meal replacement powder to make either a standard or protein-enriched meal replacement.

## Methods

The study protocol was approved by the University of California Los Angeles Institutional Review Board. Healthy volunteers were recruited by public advertisement. Subjects over 30 years of age with a body mass index (BMI) between 27 to 40 kg/m^2^, and in good health by history, physical examination, and basic laboratory screening (complete blood count, serum chemistries, liver panel, and lipid panel) were selected for the study. Subjects with type 2 diabetes or glucose intolerance were excluded as were individuals, who regularly drank more than one alcoholic beverage daily.

One hundred men and women who met the selection criteria were randomly assigned to either the HP (high protein) or SP (standard protein) treatment. This was a single-blinded study. Subjects were randomized in a 1:1 manner to either HP or SP diet using a computerized random proportion model.

Caloric intake to achieve weight loss was based on a 500 Kcal deficit of the participants' estimated resting metabolic rate as determined by body composition analysis by DEXA. Diet plans were individualized per subject by the research dietitian. Subjects were instructed to add to their meal replacements a set number of scoops of either protein or carbohydrate from powder canisters labeled as either A or B. The protein powder was measured with a calibrated scoop and subjects were instructed regarding how many scoops to use for their particular meal plan. Participants in the HP group received a diet plan that provided 2.2 grams of protein per kg of LBM while the diet for the SP group provided 1.1 grams of protein per kg of LBM. The meal energy macronutrient composition in the HP group was approximately 30% protein, 30% fat, and 40% carbohydrate. The macronutrient composition in the SP diet was approximately 15% protein, 30% fat, and 55% carbohydrate. Both groups received the same isocaloric MR (Formula 1, Herbalife Intl., Los Angeles) with either a protein supplement for the HP group (Performance Protein Powder, Herbalife Intl., Los Angeles) or a carefully matched carbohydrate placebo containing maltodextrin and flavoring for SP group.

Instructions were provided for preparation of the MR. Subjects were advised to consume one MR in place of a meal and the other as a snack daily for 12 weeks, then one MR a day for an additional 40 weeks. All participants met individually with a registered dietitian at baseline for dietary instruction, and at week 2, month 1, 2, 3, 6, 9 and 12 to provide counseling and follow-up. Qualitative food logs including the servings of macronutrients and meal replacements were collected and reviewed with subjects at each visit. Participants were weighed and protein powder meal replacement products were dispensed at each visit. Subjects were given general advice for increasing their activity level with a goal of 30 minutes of aerobic exercise per day.

### Body Weight

Subjects were weighed at each visit (Detecto-Medic; Deteco-Scales; Brooklyn, NY) while wearing no shoes after an overnight fast. Height was measured with a stadiometer (Detecto-Medic; Deteco-Scales; Brooklyn, NY) at week 0. BMI was calculated as weight (kg)/height squared (m).

### Biochemistry

Blood samples after >10 hours of overnight fasting were collected at months 0, 3, 6, and 12 for measurement of lipid profiles, electrolytes, liver and renal function tests. Twenty-four hour urine samples were collected at baseline and week 52 for urinary urea nitrogen, creatinine, calcium, phosphate excretions.

Plasma cholesterol was determined using standard enzymatic methods. Reagents, standards and calibrators were purchased from Pointe Scientific (Lincoln Park, MI). The HDL or alpha cholesterol is derived from the measurement on the supernatant following the precipitation of apo B containing lipoproteins with Heparin and MnCl_2_. The so-called LDL or beta lipoprotein cholesterol is estimated from these data using the Friedewald equation. All other tests were completed at Ronald Reagan Medical Center clinical laboratory using standard methods. Urinary urea nitrogen was measured with an enzymatic method of Talke and Schubert [13].

### Bone Density

Bone density was measured at baseline and 12 months by Dual Energy X-ray Absorptiometry by a Lunar Prodigy DEXA (GE Medical Systems, Waukesha, Wisconsin).

### Statistical Analysis

All variable transformations and statistical analyses were performed using SAS version 9.2 [14]. We evaluated effectiveness of the subject random allocation by comparing patient characteristics and baseline measurements of the two study groups using t-tests (for continuous variables) and Chi-square tests (for categorical variables).

We computed t-tests within each treatment group using matched pair Analysis of Variance (ANOVA). Univariate and multivariate Repeated Measures ANOVA described within subject effects of changes over time for the total study sample; between treatment group effects; and changes over time by treatment group interactions. Because outcome data was not available for participants who dropped out of the study, we did not conduct intention to treat analysis. All data are presented as means ± standard deviation of the mean (SD).

## Results

100 obese men and women were randomly assigned to either a HP or SP MR diet plan. Fifteen subjects withdrew from the study within the first week after randomization due to inability to comply with the meal plan (6 in the HP group and 9 in the SP group) and those subjects were excluded from data analysis. Fifteen more subjects (9 in the HP group and 6 in the SP group) dropped out of the study during the 12 month trial due to loss of follow-up and personal reasons. No subject suffered any severe adverse event. Seventy subjects, (thirty-five subjects in each group) completed the 12-month study. Subject characteristics in the two treatment arms at baseline were not significantly different (Table 1). Mean age was 49.4 ± 11.0 years. Mean BMI at baseline was 34.43 ± 6.36 for HP group and 32.57 ± 4.10 kg/m^2 ^for SP group.

**Table 1:** Baseline characteristics of study participants

Characteristic		HP (N = 44)	SP (N = 41)
Demogrphic			
Women, No. (%)		36 (81.8)	26 (63.4)

Age, mean (SD)		48.9 11.8)	49.7 (9.1)

Race, No. (%)			
	Asian	4 (9.1)	1 (2.4)
	Black	9 (20.5)	8 (19.5)
	Caucasian	26 (59.1)	28 (68.3)
	Hispanic	4 (9.1)	2 (4.9)
	Others	1 (2.2)	2 (4.9)

Weight Factors, mean (SD)			
Body Weight, kg		93.5 (14.0)	92.7 (15.9)

BMI, kg/m2		34.7 (6.8)	34.3(10.3)

All values are mean ± SD. Subjects who dropped out of the study after randomization were excluded. There were no significant differences between groups. HP: high protein group; SP: standard protein group.

### Weight Loss

**Figure 1:**
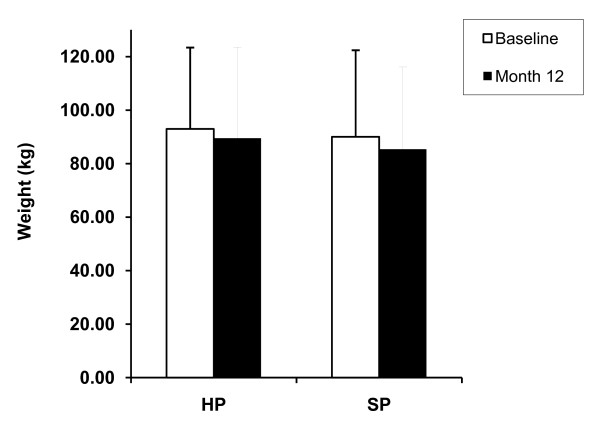
**Body weight change in 12 months**. Mean ± SD. Subjects in HP and SP group both lost significant amount weight in 12 months compared with baseline. Open square: baseline, black square: month 12, *P < 0.01 compared with baseline

Subjects were weighed at baseline, 2 weeks, and monthly thereafter. Baseline body weight was not significantly different between these two groups. Both groups lost significant amounts of weight at 12 months (4.29 ± 5.90 kg; SP -4.66 ± 6.91 kg, p < 0.01). (Figure 1) After controlling for baseline weight, gender, and time period, there was no significant difference between the two treatment groups. For both dietary groups, BMI was significantly lower at 12 months (HP = -1.53 ± 2.17; SP = -1.77 ± 2.89 kg/m^2^). There were no significant differences in BMI changes between the two dietary groups.

### Cholesterol, HDL, LDL, triacylglycerol

There were significant reductions in total cholesterol for the HP group at 3 months (-15.20 ± 35.84 mg/dL, p < 0.05) and 6 months (-10.47 ± 30.46 mg/dL, p < 0.05) but not for the SP group (-4.98 ± 25.14; -9.31 ± 30.26 mg/dL, p > 0.05). The LDL concentration was significantly lowered at 3 months and 6 months ( -7.74 ± 21.92; -7.83 ± 23.06 mg/dL, p < 0.05) for the HP group but not for SP group. There was significant elevation of HDL at month 6 for HP group only (2.53 ± 7.45 mg/dL, p < 0.05). The triacylglycerol concentration was reduced significantly only for the HP group at 3 months (-29.73 ± 58.22 mg/dL, p < 0.05). The difference between the two groups was not significant for any of the parameters. (Table 2)

**Table 2:** Blood lipid concentrations

	Cholesterol (mg/dL)		Triacylglycerol (mg/dL)		LDL (mg/dL)		HDL (mg/dL)	
	
	HP	SP	HP	SP	HP	SP	HP	SP
Baseline	198.85 ± 311.00	203.04 ± 39.08	136.07 ± 105.61	115.16 ± 55.57	116.88 ± 35.92	128.07 ± 36.28	54.34 ± 13.82	52.00 ± 10.56
	
Month 3	184.93 ± 272.00*	196.68 ± 36.36	106.24 ± 52.66*	113.59 ± 63.17	108.88 ± 36.54*	121.29 ± 36.36	54.83 ± 15.11	52.73 ± 11.19
	
Month 6	190.71 ± 279.00*	195.05 ± 38.38*	116.74 ± 74.19	106.64 ± 52.44	111.73 ± 26.79 *	119.74 ± 34.86 *	56.47 ± 15.47 *	53.95 ± 11.77
	
Month 12	188.33 ± 292.00	201.24 ± 37.60	119.33 ± 53.41	109.42 ± 63.78	132.07 ± 34.09	125.33 ± 32.78	54.63 ± 13.48	54.00 ± 11.48

All values are mean ± SD. There were no significant differences between groups. HP, high protein group; SP, standard protein group.

* p < 0.05 compare with baseline

### Liver function

All subjects had normal ranges of AST, ALT, bilirubin, and alkaline phosphatase at baseline. All those markers remained in the normal range and did not change significantly through the study (Table 3). No subject had any liver markers out of the normal range during any time of the study.

**Table 3:** Liver function tests

	ALT (U/L)		AST (U/L)		Alkaline Phosphatase (U/L)		Total Billirubin (mg/dL)	
	
	HP	SP	HP	SP	HP	SP	HP	SP
Baseline	27.07 ± 13.97	28.84 ± 15.00	25.2 ± 11.44	23.33 ± 13.00	66.2 ± 37.00	71.88 ± 41.00	0.75 ± 0.26	0.77 ± 0.40
	
Month 3	25.4 ± 7.51	28.53 ± 15.00	24.23 ± 7.30	24.39 ± 16.00	69.64 ± 39.00	72.85 ± 18.58	0.75 ± 0.20	0.82 ± 0.20
	
Month 6	24.87 ± 11.22	27.23 ± 16.00	24.26 ± 14.00	23.44 ± 5.17	69.89 ± 19.13	72.21 ± 17.78	0.80 ± 0.25	0.83 ± 0.30
	
Month 12	24.1 ± 12.00	26.91 ± 14.23	23.00 ± 13.00	23.76 ± 14.00	71.48 ± 40.00	69.18 ± 18.65	0.76 ± 0.36	0.84 ± 0.40

All values are mean ± SD. There were no significant differences between groups. HP, high protein group; SP, standard protein group.

### Renal function

No significant differences were found when comparing 12 month mean concentrations of serum creatinine, urea nitrogen and urine nitrogen and creatinine clearance within the groups and between the groups (Table 4). Urinary protein excretion significantly increased in the SP group but not in the HP group at month 12 (HP: 27.18 ± 105.33, mg/24 hours, p = 0.410; SP: 54.82 ± 83.35 mg/24 hours, p = 0.02). There was not any difference between the groups.

**Table 4:** Renal function, calcium, phosphate excretion and bone mineral density.

	HP		SP	
	
	Baseline	Month 12	Baseline	Month 12
Serum Creatinine (mg/dL)	0.82 ± 0.20	1.13 ± 1.85	0.87 ± 0.20	0.82 ± 0.18
Serum urea nitrogen (mg/dL)	12.37 ± 3.06	14.13 ± 5.77	12.14 ± 3.77	11.97 ± 3.73
Creatinne Clearance (mL/min)	129.78 ± 60.06	138.69 ± 40.39	116.89 ± 44.43	116.89 ± 42.84
Urine urea nitrogen (g/24 hr)	10.91 ± 4.49	12.22 ± 4.64	10.89 ± 4.73	9.58 ± 3.95
Urine Calcium (mg/24 hr)	184.68 ± 119.10	153.46 ± 77.07	25.2 ± 103.60	23.33 ± 75.74
Urine Protein (mg/24 hr)	141.25 ± 71.23	158.55 ± 88.82	114.39 ± 38.25	180.00 ± 86.56*
Bone mineral density (g/cm^2^)	1.00 ± 0.00	1.04 ± 1.19	1.03 ± 0.17	1.01.00 ± 0.03

All values are mean ± SD. There were no significant differences between groups. HP, high protein group; SP, standard protein group. *p < 0.05 compare with baseline

### Bone mineral density

No significant differences (p > 0.05) were observed at 12 months in total bone mineral density within-group or between groups (Table 3).

## Discussion

In this study, the energy deficit meal plan including meal replacements resulted in significant weight loss typical of meal replacement plans in both groups [15]. Since there was no run-in period, early dropouts were significant but 70 out of 85 subjects were retained after that point of the study. Because both diets were isocaloric the amounts of weight loss were the same enabling a meaningful comparison of the effects of the dietary intervention on liver function, renal function, and bone density in an outpatient setting. No special efforts were made to assess compliance which could be considered a limitation of the study. Compliance with diets is known to decrease on an outpatient basis and is an unmeasured effect that may account for the lack of findings of adverse events in our study. Nonetheless, this was a practical applied test of the issue as it would be encountered in people undertaking a weight management regimen.

Concerns that diets high in protein may have deleterious effects on renal function were not supported by the results of this study. There was no difference in creatinine clearance with either dietary pattern during weight reduction over one year. A previous study also reported that creatinine clearance was not altered by dietary protein in the context of weight loss while nitrogen balance was more positive in subjects who consumed a high protein diet than in those who consumed a high carbohydrate diet [16]. Skov et al [8] assessed changes in renal function by measuring the glomerular filtration rate (GFR) during high-protein and high-carbohydrate diets over a 6-month period and found that the high protein diet had no adverse effects on kidney function. More recently, Knight et al. determined whether protein intake influences the rate of renal function change in women prospectively studied over an 11-year period [7]. The Nurses' Health Study evaluated 1624 enrolled women between the ages of 42 to 68 years in 1989 who provided blood samples in 1989 and 2000. Ninety-eight percent of women were white, while 1% were African American. In multivariate linear regression analyses, high protein intake was not significantly associated with change in estimated GFR in women with normal renal function (defined as an estimated GFR ≥ 80 mL/min per 1.73 m^2^).

It has been suggested that a high protein diet may generate acidosis because of the presence of ketone bodies in the blood promoting calcium mobilization from bone to buffer the blood and maintain pH. This could promote urinary calcium loss [17,18]. There were no deleterious effects of increased protein intake at 2.2 g/kg LBM on markers of bone turnover in our study. In a 12- week study [19], a high protein diet increased the bone turnover markers while calcium excretion was decreased by 0.8 mmol/d. Evidence also indicates that high protein intake particularly higher animal protein intake is associated with decreased bone loss in older persons [20].

The trend of reduction in urinary calcium in this study was also unusual because dietary protein metabolism is associated with increased urinary calcium [21]. The high vegetable consumption with both dietary patterns may prevent this because high vegetable intakes have been shown to decrease urinary calcium [22]. An increase in calcium excretion was observed with the consumption of a high protein diet in the study by Johnston et al [16] which stated that this was due to the high calcium content of the high protein diet in their study. However, we did not observe this high protein pattern in which dietary calcium was very high.

Non-alcoholic fatty liver disease (NAFLD) is now the most common liver disease and is strongly linked to obesity and metabolic syndrome [23]. In middle aged women in the UK, Liu and colleagues [24] found that the relative risk of liver cirrhosis increased by 28% for every 5 unit increase in BMI above 22.5 in each stratum of alcohol consumption and estimated 17% of incident or fatal liver cirrhosis is attributable to excess body weight. Hart and colleagues [25] also show that being overweight or obese and drinking alcohol has a synergistic effect, which amplifies the insult to the liver and greatly increases the risk of liver related morbidity and mortality. Therefore, it is important to demonstrate that an effective weight management program does not elevate liver function tests and add insult to the liver. In this study, there were no adverse effects on liver function tests at either level of protein intake.

The one-year duration of the study may have led to reduced compliance to the meal plans. The study subjects met for a total of 8 sessions with our dietitian. These sessions were designed to support and encourage participants to follow the meal plan including the MR. At each visit, qualitative food logs for macronutrients and meal replacement were collected and reviewed. While we did not measure biochemical compliance, the overall weight loss we observed suggest relatively good compliance to our meal plans.

Noakes et al [19] reported that subjects with high serum triacylglycerol (>1.5 mmol/L) lost more fat mass with the high protein diet than with the high carbohydrate diet and suggesting a variation in responsiveness to diet based on other metabolic factors such as the presence of insulin resistance which was not measured in the current study.

As in many outpatient diet interventions long-term compliance is undercut by some unmeasured factors likely unrelated to the demonstrated satiety effects of added protein. Therefore, the expected effects on increased weight loss resulting from a high protein diet were not seen in this study. In our previous study, protein-enriched meal led to increased fat mass loss based on bioelectrical impedance analysis in spite of similar overall weight loss as the standard protein meal plan over 12 weeks [26]. The use of MR may have been the major influence on the weight loss by simplifying their weight loss efforts so that the power of the MR intervention may have obscured the difference between the weight losses of subjects using protein-enriched meal plans by comparison to standard meal plans [5].

The Institute of Medicine (IOM) of the National Academy of Sciences [27] has set acceptable macronutrient distribution ranges for carbohydrate (45%-65% of energy), protein (10%-35% of energy), and fat (20%-35% of energy; limit saturated and trans fats). These proportions provide a range broad enough to cover the macronutrient needs of most active individuals, but specific carbohydrate and protein recommendations are also typically made based on a g/kg body weight formula. These ranges are 5 to 12 g of carbohydrate/kg body weight and 1.2 to 1.8 g/kg body weight for protein depending on the level of physical activity. Clearly, for both the HP and SP group dietary protein intakes were within this recommended range for protein intake. Therefore, our research can only be applied to structured meal plans using protein-enriched shakes for their ability to increase satiety and should not be interpreted as a blanket endorsement of very high protein diets popular with some athletes exceeding the IOM recommendations by including pure protein supplements, high fat animal meats or other sources of organic acids and hidden fat which could adversely affect liver function, renal function, or bone density.

## Conclusions

In summary, both the HP and SP diets resulted in the expected weight loss typical of an MR diet plan in free-living individuals at 12 months. Both diets were well tolerated, sustainable, and did not result in any adverse effects. There were no changes of liver function, renal function or bone mineral density based on routine clinical assessments.

## Competing interests

The authors declare that they have no competing interests.

## Authors' contributions

LT participated in the conduct of the study, the analysis of the data. SC participated in the conduct of the study. EY participated in the conduct of the study. CC participated in the study design and statistical analysis. GT participated in the study coordination. ZL conceived of the study, participated in its design and coordination, and drafting the manuscript. All authors read and approved the final manuscript.
